# Is Brain Activity during Action Observation Modulated by the Perceived Fairness of the Actor?

**DOI:** 10.1371/journal.pone.0145350

**Published:** 2016-01-28

**Authors:** Joset A. Etzel, Nikola Valchev, Valeria Gazzola, Christian Keysers

**Affiliations:** 1 Department of Neuroscience, University Medical Centre Groningen, University of Groningen, Groningen, The Netherlands; 2 University of Groningen, University Medical Center Groningen, Neuroimaging Centre, Groningen, The Netherlands; 3 Cognitive Control and Psychopathology Laboratory, Washington University in St. Louis, St. Louis, United States of America; 4 Department of Otorhinolaryngology/Head and Neck Surgery, University Medical Center Groningen, University of Groningen, Groningen, The Netherlands; 5 Social Brain Lab, Netherlands Institute for Neuroscience, Royal Netherlands Academy of Arts and Sciences, Amsterdam, The Netherlands; 6 Department of Psychology, University of Amsterdam, Amsterdam, The Netherlands; University G. d'Annunzio, ITALY

## Abstract

Perceiving other people’s actions triggers activity in premotor and parietal areas, brain areas also involved in executing and sensing our own actions. Paralleling this phenomenon, observing emotional states (including pain) in others is associated with activity in the same brain areas as activated when experiencing similar emotions directly. This emotion perception associated activity has been shown to be affected by the perceived fairness of the actor, and in-group membership more generally. Here, we examine whether action observation associated brain activity is also affected by the perceived social fairness of the actors. Perceived fairness was manipulated using an alternating iterated Prisoner’s Dilemma game between the participant and two confederates, one of whom played fairly and the other unfairly. During fMRI scanning the participants watched movies of the confederates performing object-directed hand actions, and then performed hand actions themselves. Mass-univariate analysis showed that observing the actions triggered robust activation in regions associated with action execution, but failed to identify a strong modulation of this activation based on perceived fairness. Multivariate pattern analysis, however, identified clusters potentially carrying information about the perceived fairness of the actor in the middle temporal gyrus, left postcentral gyrus, right inferior parietal lobule, right middle cingulate cortex, right angular gyrus, and right superioroccipital gyrus. Despite being identified by a whole-brain searchlight analysis (and so without anatomical restriction), these clusters fall into areas frequently associated with action observation. We conclude that brain activity during action observation may be modulated by perceived fairness, but such modulation is subtle; robust activity is associated with observing the actions of both fair and unfair individuals.

## Introduction

In humans, networks of brain regions have consistently been identified in functional magnetic resonance imaging (fMRI) studies as having “mirror” properties: activating to both the observation and execution of actions [1]. The most reliable members of the network, be it across participants [2], or across studies in meta-analyses [1, 3], are the premotor cortex, inferior frontal gyrus, primary somatosensory cortex and inferior parietal cortex, human homologues of the brain areas in which mirror neurons were originally identified in monkeys [4]. Co-localized activations have not only been found for action observation and execution, but also associated with perceiving and experiencing emotional states [5–8], as well as for both painful and painless touch [9, 10]. The current study used fMRI, and so is sensitive to BOLD responses at the scale of regions, not individual neurons. We thus do not speculate further about possible mirror neuron involvement, but intend the phrase “action observation network” to indicate brain areas containing information about the action being observed in others, and “shared circuits” to indicate regions activated both when perceiving others’ actions and when performing similar actions.

Which factors modulate how strongly people recruit brain areas involved in their own actions or emotions when perceiving the actions or emotions of others is a related topic of investigation [8, 11]. For example, the observer’s past experience, such as extensive training in a particular style of dance, can modulate activation strength [12–15], in line with the fact that action experience takes a central place in accounts of the development of the mirror neuron system (see [16] for the associative learning perspective and [17, 18] for the Hebbian learning perspective). An observer’s current motivational state [19, 20] can also modulate these activations. The degree to which an observed person is considered to belong to the same group as the observer also influences how the other’s actions and responses are perceived, the “in-group bias” phenomenon [21]. Perceived fairness is another factor known to influence activations to others’ pain. For example, Singer et al. [22] investigated whether the perceived fairness of the person in pain influenced activity in the observer’s pain network. To manipulate perceived fairness participants played a game with two confederates, one of which played a “fair” strategy, the other an “unfair” strategy. The participant was then scanned while they or the confederates experienced pain. Male participants showed less activation in their anterior insula when the “unfair” than the “fair” confederate (supposedly) experienced pain. This finding has been interpreted as a mechanism that facilitates the punishment of unfair people [23].

Here we wish to explore whether the brain activity associated with the perception of hand actions is modulated by the perceived fairness of the people observed. Akin to Singer et al. [22], our study consisted of two parts: a social manipulation and fMRI scanning. For the social manipulation the participant played an alternating iterated Prisoner’s Dilemma game with two confederates, one playing “fairly,” the other “unfairly.” During scanning the participant watched the confederates performing object-directed bimanual hand actions, as well as performed hand actions themselves. Specifically, this study tests the hypothesis that the activity in the shared circuits and action observation networks will vary depending on whether the “fair” or “unfair” confederate is in the stimuli.

## Methods

### Participants and Confederates

Sixteen healthy males ranging in age from 19 to 33 years (mean = 22.6, sd = 3.9) were recruited to participate in the experiment; one participant had excessive motion (more than 7 mm within each run) and was not analyzed. All participants reported no history of psychiatric or neurological disorders, were right-handed, had normal hearing, and normal or corrected-to-normal vision. Psychology majors were excluded to avoid participants too familiar with the Prisoner’s Dilemma game or the use of experimental confederates. Only males were included since males exhibited greater modulation of pain empathy in Singer et al. [22]. The experiment and consent procedure were approved by the Medisch Ethische Toetsingscommissie of the University Medical Center Groningen and conducted in accordance with the Declaration of Helsinki. Written consent was obtained from participants in the prescribed manner. The two confederates were both graduate students, similar to the participants in age. The same confederates were used with all participants, but their color and role (“fair” or “unfair”) was randomized, so that each confederate was in each role and color an equal number of times (see below). Confederates and participant deception were necessary for the participants to develop a strong sense of like or dislike towards the confederates, which generally does not occur if the participants know that the confederates are working for the experimenters (e.g., nonintentional findings in [24]).

### Cover Story and Preliminary Appointment

A cover story and preliminary appointment were used to reduce participant suspicion and to provide a plausible description of the experiment which explained why the participant would see the men he played the game with in the stimuli presented during scanning. The cover story was that the experiment concerned the relationship between how people play strategy games and how their brain reacts when they see other people’s actions, giving as an example of this relationship the differing ability of people to detect bluffing in poker; see the Cover Story section of S1 Text for the version used in participant recruitment emails. To strengthen the cover story, as well as familiarize the participants with the types of visual and auditory stimuli they would see during the scanning session, the participants were brought to the lab for a preliminary appointment.

The preliminary appointment was always held at least one day prior to the game and fMRI scanning session, “to ensure there is sufficient time to prepare the recordings”. The experimenter explained that all participants were recorded in the same way, and that he would see and hear others’ recordings (and others would see and hear his recordings) in the scanner. The experimenter demonstrated each action carefully, and recorded several repetitions, as if obtaining high-quality recordings were important. The participants did not perform the same actions as those presented during scanning, but were told that there were two sets of actions, which, if queried, was “necessary for randomization”. The visual stimuli were recorded first, with the participant filmed shutting a jar, flipping a coin, putting a video tape into its case, opening a notebook, and capping a memory stick, all while wearing a large green long-sleeved shirt over his clothing. Then, the sound stimuli were recorded: popping bubble wrap, clicking a mouse, stapling paper, opening hook-and-loop tape, and clapping hands. The participant was told that the experimenter would create control sounds by putting their recordings through a computer program, as remaining still (as was done for the control movies) produces no sound.

### Game Session

The game session provided social interaction between the participant and the confederates in a controlled setting in which one confederate could act fairly and the other unfairly. The game session lasted about an hour. The experimenter explained the procedures and consented all three “participants” together. The game was an alternating [25, 26] version of the Prisoner’s Dilemma (e.g. [27–29]), played face-to-face, with the participant, two confederates, and experimenter sitting around a table. A detailed description of the game, including the payout matrices, is included in the Game Session section of the S1 Text. The game session also served to familiarize the participant with each confederate’s assigned color, which was necessary since the confederates were identified by color in the stimuli used during scanning. Accordingly, the confederates and participant wore their assigned colored shirt over their clothing during the game session, the score sheets (Fig A in S1 Text) were marked by color, and the experimenter consistently referred to the players by color.

With each participant one confederate was randomly assigned to play a cooperative, “fair” strategy, while the other confederate played an aggressive, “unfair” strategy. In this version of Prisoner’s Dilemma it was not possible to assign the confederates a specific set of moves, since the partner sees the leading player’s move, and so a fixed set of moves would cause the confederates to play irrationally. Instead, the confederates played a consistent strategy, adapted as needed to each participant’s playing style. For instance, the fair confederate nearly always responded with a “C” (cooperating) when his partner led with a “C”; the unfair confederate was much more likely to respond with a “D” (defecting), and was also less forgiving of his partner’s defections. The confederates played their assigned strategy with each other, as well as with the participant; across participants, each confederate played each role equally often.

### Scanning Session

During the scanning session participants perceived (visual and auditory) and performed (executed) bimanual hand actions. The perception task always preceded the execution task, to avoid the risk that recently executing hand actions could bias activity during observation towards motor regions. Imaging was performed with a Philips Intera 3T Quaser scanner with a synergy SENSE head coil and maximum gradient strength of 30 mT/m. A sparse sampling acquisition was used during the perception runs to allow participants to clearly hear the sound stimuli. The scan cycle (TR) was composed of 25 axial slices (4.5 mm thickness, 3.5 x 3.5 mm in-plane resolution, .1 mm slice gap) collected in 1.23 seconds followed by 4 seconds of silence; stimuli (of duration 4 seconds) were presented in the silent period. Consecutive blocks were separated by 3 TR (about 16 seconds). Following the functional scanning a T1 weighted structural scan was acquired (TR = 15.31 ms, TE = 3.6 ms, and flip angle = 8 degrees).

Three scanning runs of the observation task were presented; each run contained a total of 32 blocks, divided equally between the stimulus types. Each block was made up of three stimuli of the same type (visual or auditory, action or control, fair or unfair confederate). Additionally, each participant was shown two odd-ball blocks. Detection of the odd-ball block was indicated by pushing a button with the right hand. The odd-ball was a mixed block, with either the confederate identity or stimulus type (action or control) mixed, but not modality (visual or auditory), to focus attention on both the type of action and the person performing the action. For example, an odd-ball block could consist of a control movie of the blue-shirted confederate followed by two control movies of the orange-shirted confederate. Odd-ball blocks and any blocks with a button push were omitted from further analysis.

**Fig 1:**
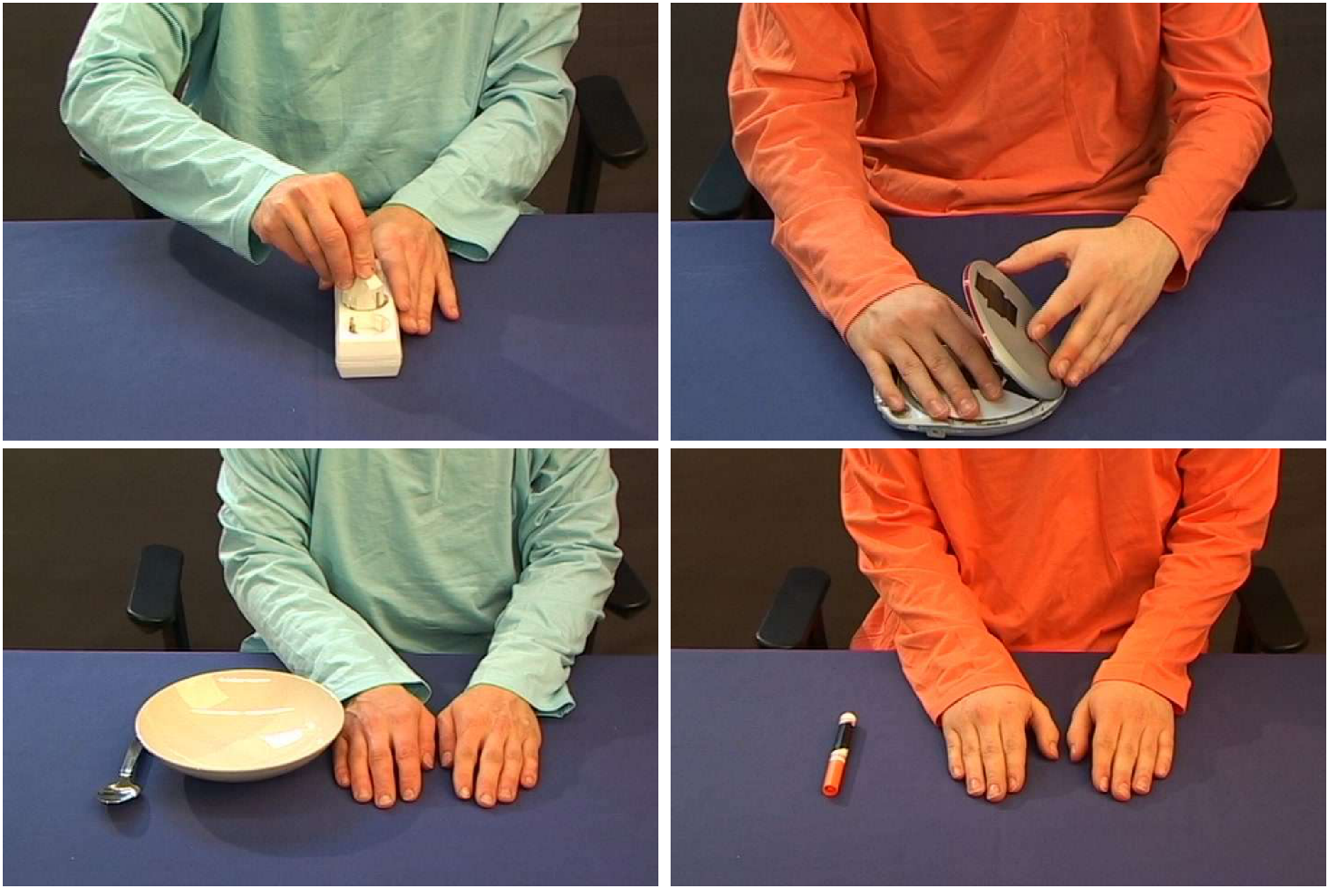
Representative frames from the action (top) and control (bottom) movies. Four of the actions are depicted here (clockwise from upper left: plugging a cord into an outlet, putting a CD into a CD player, uncapping a marker, dipping a spoon into a bowl); the fifth action (not shown) was taking a tea bag out of a cup.

The movie stimuli (Fig 1) showed the confederates, wearing the same colored shirts as in the participant’s game session, performing bimanual hand actions: uncapping a marker, dipping a spoon into a bowl, putting a CD into a CD player, plugging a cord into an outlet, picking a tea bag out of a cup. Control movies showed the confederates remaining motionless, their hands flat on the table beside the objects. The sound stimuli were a subset of those from Gazzola, Aziz-Zadeh et al. [30]: the five action sounds (ripping paper, unrolling adhesive tape, opening a zipper, opening a soft drink can, and crushing a soft drink can), with the unrecognizable phase-scrambled versions of each sound as controls. Although the participants were told that the sound clips were recorded from each confederate, only one set of sound clips were used. The confederate performing the action could be seen in the movies by the color of his shirt and shape of his hands, but to minimize visual input during the sound stimuli we identified the (supposed) confederate creating each sound by a colored square, which appeared 1.7 seconds before the start of each sound block and remained visible throughout the block. Thus, if confederate A wore the blue shirt during the game session, the participant would see confederate A wearing the blue shirt in the movie stimuli during the scanning session, and see a blue square during the sound clips supposedly recorded from confederate A.

The bimanual hand action execution task consisted of the same procedure and actions used in Gazzola, Aziz-Zadeh et al. [30]. Briefly, the participant viewed a red cross, and when it changed to green he raised his hands and received either a peanut (which he broke in half) or a piece of paper (which he tore in half) from the experimenter. The cross returned to red after 4 seconds, at which time the participant stopped the action and returned to the rest position. The objects were presented in one random order in a single run, with eight repetitions of each object. A continuous acquisition sequence was used (TR = 2s, 39 axial slices, 3.5 mm slice thickness, 3.5x3.5 mm in-plane resolution, 0 mm slice gap).

### Image Preprocessing and First Level Analysis

Image preprocessing and first level analyses were carried out shortly after data collection, using SPM2 (Wellcome Department of Imaging Neuroscience, London, UK), which was in wide use at the time (2008). Image preprocessing consisted of motion correction and spatial normalization to the MNI template (resampling to 2x2x2 mm voxels), but not slice-time correction. Finally, and for the mass-univariate analyses only, the preprocessed EPI images were smoothed with a 6 mm FWHM Gaussian kernel.

The first level analyses were performed following SPM conventions, and using its canonical hemodynamic response function. Separate design matrixes were created for perception and execution, each with the motion parameters as covariates of no interest. Blocks containing an error, odd-ball, or button press were modeled separately and omitted from subsequent analyses. The perception runs were modeled with separate predictors for the blocks of each stimulus type (action and control; auditory and visual) and confederate. The execution run was modeled with a single predictor coding the periods of movement (the two different hand movements were modeled together). Both types of data were high-pass filtered with a filter calculated at 110% of the maximum stimulus onset asynchrony between blocks of the same type.

The first level analyses were similar when preparing images for the MVPA, except that the unsmoothed images were used, and each stimulus block was modeled separately (rather than by run). The resulting parameter estimate (beta weight) images for each stimulus block were centered (to an across-voxels mean of zero), and then averaged by stimulus type, omitting examples at random when needed for balance, resulting in a single (averaged) image for each participant and stimulus type. Subsetting prior to averaging ensures that differences in the number of images in each class will not bias the classification.

### Second Level Mass-Univariate Statistical Analyses

SPM8, with improved statistical (though not preprocessing) functions, was available when this dataset was revisited in 2014, and so the second-level mass-univariate statistical analyses were carried out in SPM8. For each first-level contrast (each individual predictor against baseline, action vs. control, and action vs. control separately for each confederate role), a one sample t-test (versus zero) was computed. Activations were thresholded at p < .05 with FDR voxel-wise multiple comparisons correction and minimum cluster size 10 voxels, unless otherwise specified. All local maxima are reported following the SPM8 Montreal Neurological Institute (MNI) anatomical template. Coordinates were attributed to specific cytoarchitectonic areas using the SPM Anatomy toolbox [31, 32].

Shared voxels were defined at the second level by contrasting the BOLD responses when perceiving action and control stimuli (at p < .05 FDR corrected), with the analysis limited to voxels whose BOLD response had been identified as significant (at p < .001 uncorrected, minimum cluster size 10) during the action execution task. This procedure defines shared voxels as voxels active both when the participant perceived and executed actions. Shared voxels were mapped in two different ways: for all stimuli together and when separating the stimuli by confederate (to allow comparisons between the shared voxels resulting from stimuli depicting the fair and unfair confederate). This definition of shared voxels requires inference at the voxel level, and hence dictates the use of voxel-wise rather than cluster-wise FDR.

### Multivariate Pattern Analysis (MVPA)

Linear support vector machines (SVM) were used for all classification analyses (c = 1; e1071 R interface to libsvm), partitioning on the participants (leave-one-subject-out; fifteen-fold cross-validation), and accuracy computed by averaging across the cross-validation folds. Between-subjects analysis was necessary because confederate identity, color, and role were confounded *within* each participant: successful classification of confederate role within a single participant could be due to a confounded factor, such as the confederates’ shirt color. However, confederate identity, color, and role were balanced *across* participants: successful between-subjects role classification involves training and testing datasets in which the fair and unfair roles are played by each confederate wearing both colors, so classification can’t depend on these confounded factors. Thus, successful between-subjects role classification suggests that the participants’ voxel-level activity patterns differed with the role of the confederate in the stimulus, and that this difference was similar in all participants.

Two complementary voxel selection techniques were employed for feature selection: searchlight analysis [33] and anatomical ROI-based analysis [34]. The role searchlight analysis used three-voxel radius searchlights (6 mm, 263 voxels maximum), and so targeted relatively focal informative areas. Large areas with weakly informative voxels are sometimes more properly identified in a region of interest (ROI) than a searchlight analysis [35, 36], so we defined three anatomical ROIs, separately in each hemisphere (Table 1, Fig B in S1 Text). The ROIs encompass core action observation regions: the premotor (preM), primary somatosensory (S1), and parietal (PFhIP) cortices [2, 3, 37–39]. The maximum probability maps based on the probabilistic cytoarchitectonic maps in the SPM Anatomy Toolbox (version 1.8, [31, 32]) were used to create the ROIs. All voxels in each ROI or searchlight were used for classification, without further feature selection.

**Table 1:** The number of voxels (2x2x2 mm) and classification accuracy of each ROI. The p-values are from the permutation test, ** p < .01 * p < .05, uncorrected. Role Accuracy is from classifying subtracted images (moving-movies—still-movies, voxelwise). The ROIs are shown in Fig B in S1 Text.

ROI	Voxel Count	Role Accuracy	Stimulus Type Acc.	
			Visual	Auditory
PFhIP, Left	3485	.5 (.57)	.73 (.004)**	.5 (.54)
PFhIP, Right	3386	.73 (.006)**	.82 (.001)**	.63 (.04)*
preM, Left	5565	.53 (.42)	.9 (.001)**	.55 (.28)
preM, Right	5290	.53 (.41)	.83 (.002)**	.48 (.66)
S1, Left	2976	.6 (.16)	.9 (.001)**	.38 (.97)
S1, Right	3190	.43 (.82)	.9 (.001)**	.5 (.57)

PFhIP: Parietal cortex and intraparietal sulcus; SPM Anatomy Toolbox (1.8) areas PFt, PFm, PFcm, PFop, hIP1, hIP2, and hIP3.

preM: Premotor cortex; SPM Anatomy Toolbox (1.8) areas BA44 and BA6.

S1: Primary somatosensory cortex; SPM Anatomy Toolbox (1.8) areas BA1, BA2, BA3a, and BA3b.

Significance levels for both the ROI-based and searchlight analyses were calculated following the permutation scheme recommended in Etzel [40] for designs in which partitioning is on the subjects. 1000 random relabelings were calculated, so .001 is the most significant p-value possible. Bonferroni multiple-comparisons correction (for six ROIs) is appropriate for the ROI-based, but not the searchlight analysis; see the MVPA Significance Testing section of the S1 Text for additional discussion. Rather than attempt an arbitrary and possibly misleading correction, we present the searchlight results uncorrected for multiple comparisons, fully acknowledging that correction would be preferred.

## Results

### Behavioral Manipulation

The game was intended to cause the participants to form opinions of the personalities of the two confederates: that one was fair and cooperative and the other unfair and uncooperative. The success of this manipulation was determined from responses to a questionnaire and to questions during debriefing. The participants’ responses (Table B in S1 Text) indicate that the manipulation was successful: all rated the fair confederate as more “pleasant and agreeable” and more “reasonable” than the unfair; no participant rated the fair confederate as less “fair” or “liked” than the unfair confederate (two participants gave the confederates equal ratings on these questions). Early in debriefing the participants were prompted for their opinions of the confederates, and these responses were also consistent with the manipulation. For example, participants described the fair confederate as “honest”, “cooperative”, and “kind”, but the unfair confederate as “antisocial”, “sly”, and “going for the win”. The last stage of debriefing consisted of informing the participant that the other “participants” were actually confederates, and the reason for the deception. Most participants expressed surprise that the other people were confederates; two reported being somewhat suspicious (one of both confederates, the other only of the “fair” confederate), but not sure.

Most participants identified at least one of the two odd-ball blocks, indicating that they attended to the stimuli. Seven participants correctly identified both odd-ball blocks (one of these participants also had a single false alarm; three had two). Five participants correctly identified one of the odd-ball blocks, of which two had additional button presses (one participant had one false alarm, the other had seven). Three participants did not identify either odd-ball block, and had no false alarms.

### Activity Associated with Action Perception

**Fig 2:**
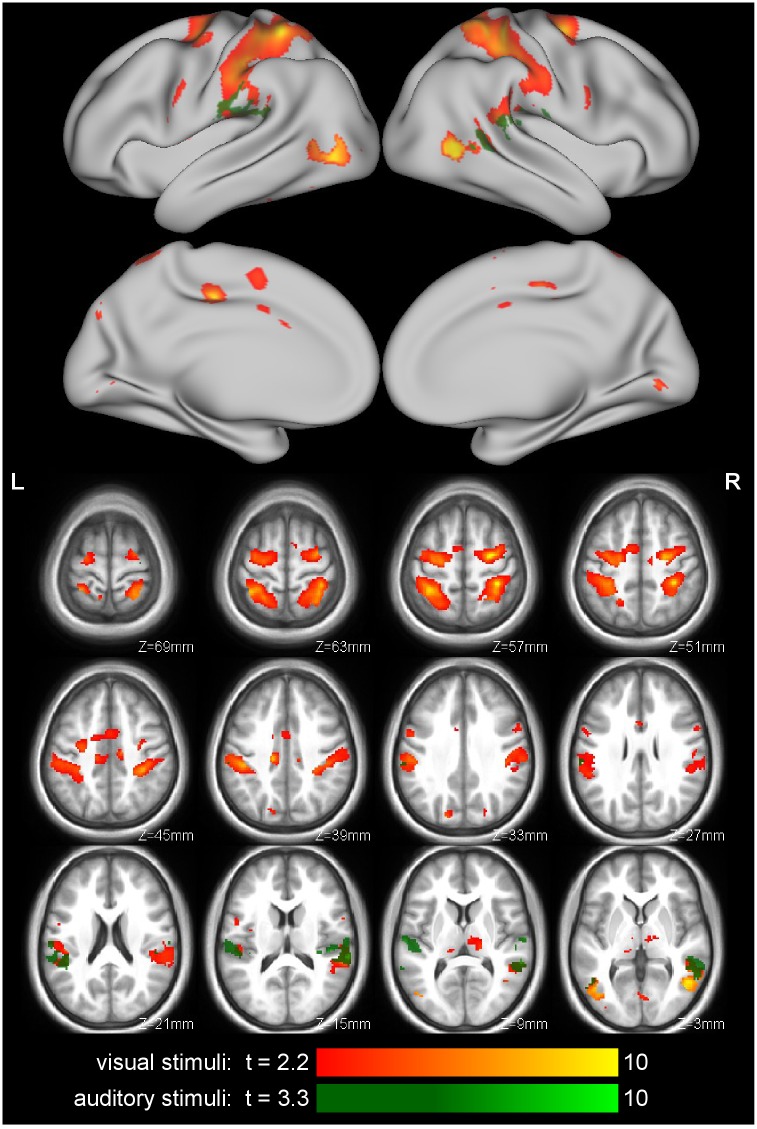
Identification of the shared circuits, by means of the mass-univariate GLM. Shared voxels resulting from the contrast (Action Movies)–(Control Movies), limited to the execution mask (p < .05 FDR correction, minimum cluster size 10 voxels) are shown in orange, with cluster sizes and coordinates are listed in Table 2. Shared voxels for the auditory stimuli, (Action Sounds)—(Control Sounds), (p < .05 FDR correction, minimum cluster size 10 voxels), limited to the execution mask are shown in green.

**Fig 3:**
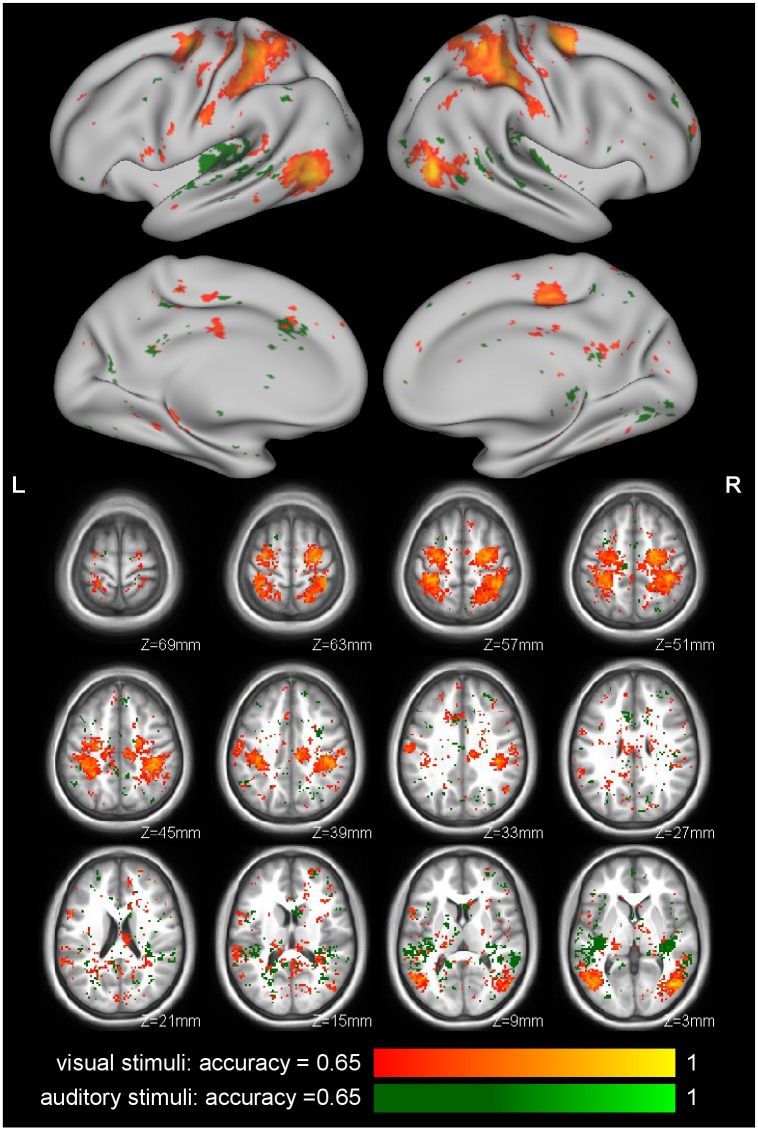
Action perception-specific responses identified by the MVPA searchlight analysis (2-voxel radius). Classification (leave-one-subject-out cross-validation) accuracies are shown for visual (orange) and auditory (green) stimuli. The visual stimuli resulted in high-accuracy searchlights in typical action observation regions, while information about the auditory stimuli was largely confined to auditory regions. For display, the classification accuracy map was thresholded at .65 and clusters of at least 20 voxels.

The shared circuits for the visual stimuli identified by the mass-univariate analysis (Fig 2 and Table 2) are similar to those found in previous studies [2, 41–45], including activation of premotor (BA44 and BA6), somatosensory (BA1 and BA2) cortices, the anterior section of the inferior parietal lobule, and the middle temporal gyrus. The MVPA classified stimulus type (action vs. control movie) with both ROI and searchlight analyses (Table 1, Fig 3), using images from the action observation scanning runs only (we did not attempt to classify action execution stimuli). The location of the peak areas from the MVPA are quite similar to the shared voxels from the mass-univariate analysis; see Fig D in S1 Text.

**Table 2:** Clusters of shared voxels (2x2x2 mm) for the visual modality. All clusters of at least 10 voxels are listed (p < .05 with FDR correction, t > 2.2). Contrast is limited by an explicit mask from the execution run (p < .001 uncorrected, minimum cluster size 10). Coordinates of the peak voxel activation, Brodmann and anatomical localization given according to the SPM anatomy toolbox.

Size (voxels)	Brodmann Area	Hemisphere	Anatomical Area	x	y	z	t
3279	BA 2	R	Postcentral Gyrus	36	-38	58	11.03
	hOC5	R	Middle Temporal Gyrus	48	-66	0	10.55
	BA 7	R	Superior Parietal Lobule	32	-54	62	6.79
2808	BA 7	L	Superior Parietal Lobule	-36	-46	60	9.86
	BA 2	L	Inferior Parietal Lobule	-44	-30	40	7.77
	BA 1	L	Superior Parietal Lobule	-30	-44	68	7.01
	BA 7	L	Precuneus	-14	-58	68	6.11
		L	SupraMarginal Gyrus	-54	-24	36	5.91
		L	Rolandic Operculum	-44	-28	20	4.78
1406	BA 6	L	Precentral Gyrus	-24	-14	56	6.46
		L	SMA	-14	-2	50	4.44
	BA 6	L	Middle Cingulate Cortex	0	2	44	3.72
		L	Anterior Cingulate Cortex	-2	12	28	3.25
	BA 6	L	Superior Frontal Gyrus	-18	-8	72	3.00
906	BA 6	R	Superior Frontal Gyrus	30	-8	58	11.08
	BA 6	R	Middle Cingulate Cortex	14	-18	48	5.51
331	hOC5	L	Middle Occipital Gyrus	-44	-72	0	8.98
		L	Middle Temporal Gyrus	-46	-66	6	8.64
182		R	Cerebelum (VI)	26	-52	-22	4.59
		R	Fusiform Gyrus	24	-60	-16	3.44
133		R	Thalamus	18	-24	10	3.96
133	BA 17	R	Linual Gyrus	4	-76	-4	4.80
117	BA 44	L	Rolandic Operculum	-50	2	14	3.08
115		L	Cerebelum (VI)	-28	-54	-22	4.97
82	BA 44	R	Precentral Gyrus	60	10	32	3.35
41		L	Cuneus	-16	-84	34	4.94
		L	Superior Occipital Gyrus	-14	-80	40	3.31
32	BA 6	R	SMA	10	0	68	2.55
27		L	Thalamus	-14	-28	8	2.97
22		L	Inferior Occipital Gyrus	-46	-62	-12	3.06
18	OP 3	L	Rolandic Operculum	-40	-6	14	2.57

The same mass-univariate shared-circuits analysis on the auditory data identified a different set of areas (Fig 2): the auditory cortex, with activations extending into the anterior inferior parietal lobe, but not premotor cortices. Similarly, the MVPA (Table 1, Fig 3) found discrimination between action and control sounds in the auditory cortex, and posterior insula, but little in more dorsal regions. This difference between responses to visual and auditory stimuli may be due to task demands: participants were not asked to recognize the body part creating the action sounds (e.g. by the mouth or hand as in Gazzola et al. [30]), but rather to identify within-block stimulus consistency (type and confederate identity). Indeed, since confederate identity was indicated by a colored square, the current version of the task diverted attention away from auditory information and may thus have reduced action specific activity. We therefore decided not to include the auditory data in the later analyses.

### Activity Associated with Confederate Role: Fair vs. Unfair

**Fig 4:**
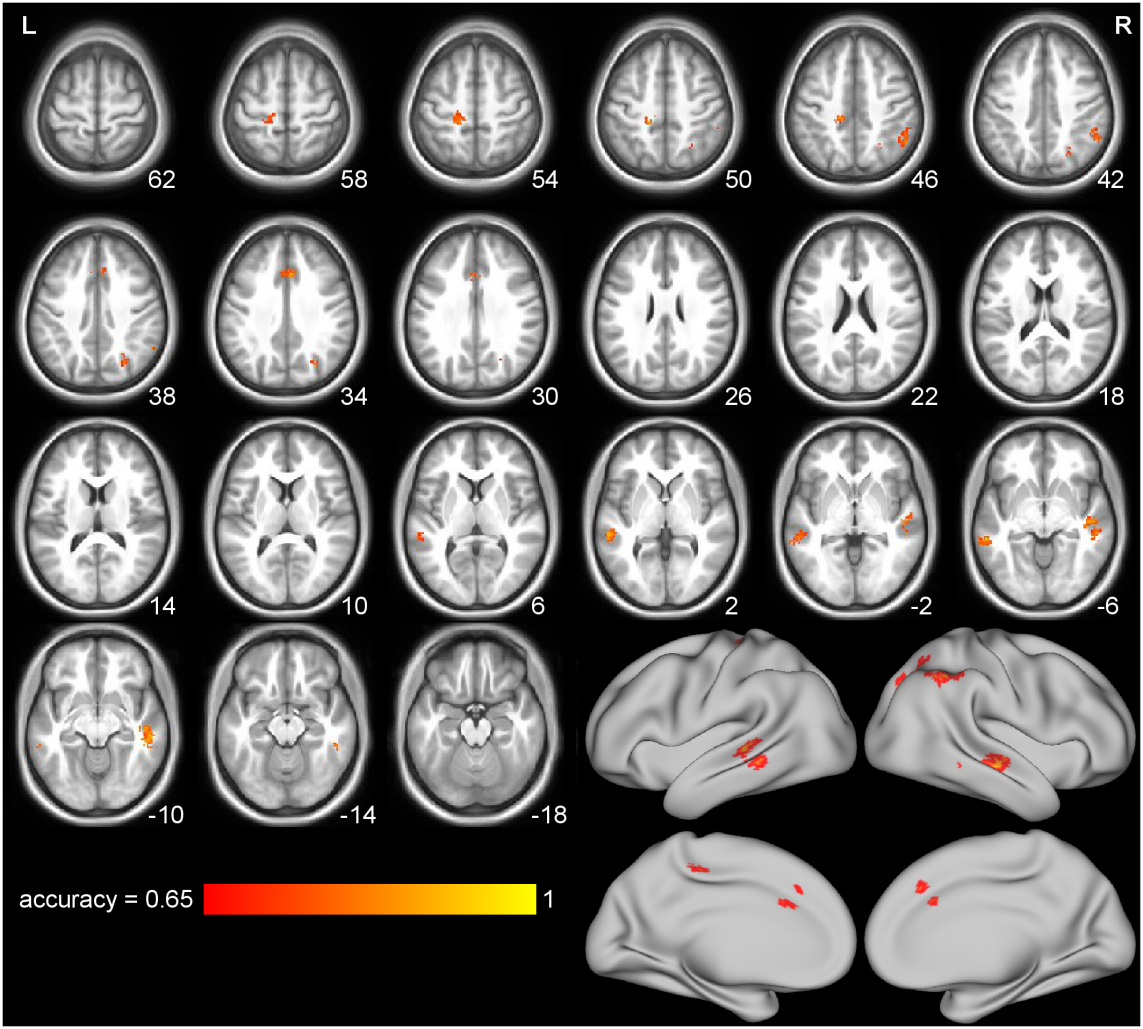
The six clusters in which confederate role could be classified in the searchlight MVPA. Slice numbers are coordinates in mm. These clusters were identified by taking voxels with p < .01 in the permutation test, with a minimum cluster size of 50 voxels; Fig E in S1 Text has a less thresholded (p < .05 uncorrected) image for comparison.

These analyses test whether activity in the shared circuits in particular, or the action observation network more generally, is modulated by the perceived fairness of the people being observed. In the MVPA we addressed this question by examining whether action-specific brain activation patterns (generated by subtracting the mean PEI for the control movies from the mean PEI for the action movies, separately for each confederate and participant) could be classified according to confederate *role*. Classification of confederate role was possible using MVPA in the right PFhIP ROI (Table 1) associated with action observation, as well as in six clusters in the searchlight analysis (Fig 4, Table 3, Fig E in S1 Text), treating voxels with p < .01 in the permutation test and in clusters of least 50 voxels as significant. While we interpret the areas shown in Fig 4 as containing information about the fairness of the actor as well as the observed action, the effect is not particularly robust: the informative areas are a small fraction of the action observation network found previously (Fig 3), and successful classification is sensitive to analytical choices (e.g., role cannot be classified if the images for the control and action movies are analyzed separately rather than after subtraction).

**Table 3:** Size, location, and description of the six clusters in which confederate role could be classified in the searchlight MVPA; see Fig 4. Coordinates of the center of mass, Brodmann and anatomical areas given according to the SPM Anatomy Toolbox.

Size (voxels)	Brodmann Area	Anatomical Area	x	y	z
237		Right Middle Temporal Gyrus	52	-32	-10
		Right Superior Temporal Gyrus	52	-20	-4
192		Left Middle Temporal Gyrus	-56	-34	0
126	3a		-16	-34	52
	4p	Left Postcentral Gyrus	-22	-32	56
	4a		-12	-26	58
106	PFm	Right Inferior Parietal Lobule	52	-48	44
81		Right Middle Cingulate Cortex	6	22	34
66	hIP3	Right Angular Gyrus	26	-60	48
	7a	Right Superior Occipital Gyrus	28	-64	40

**Fig 5:**
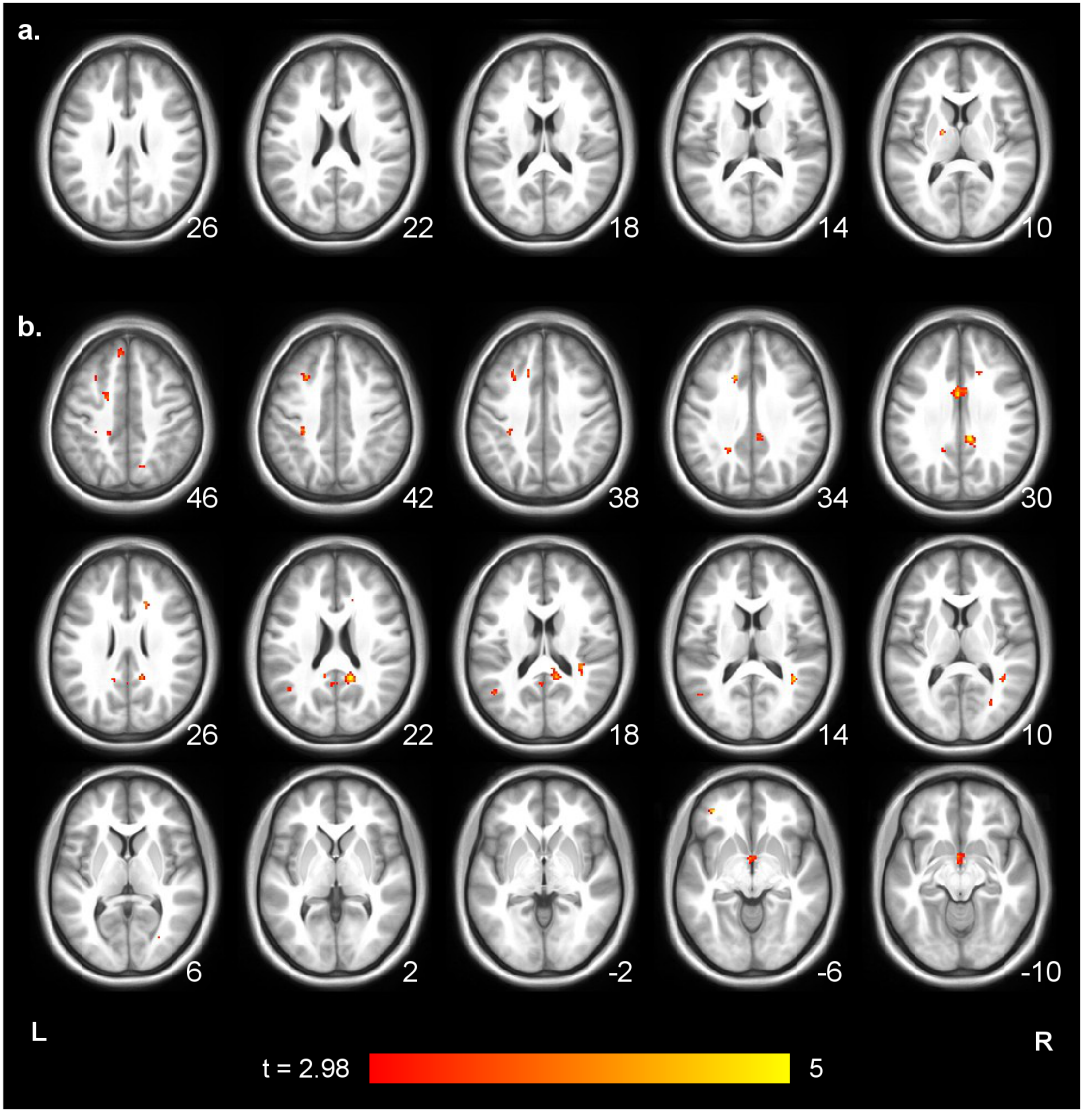
Axial slices showing the activated voxels for the contrasts. a) (Action Movies—Control Movies) fair—(Action Movies—Control Movies)unfair, not masked with execution and b) (Action Movies—Control Movies)unfair—(Action Movies—Control Movies) fair not masked with execution. Both thresholded at p < .005 uncorrected (t>2.98), min cluster size 10 voxels.

Formulated in mass-univariate (GLM) terms, the target analysis was to identify modulation of the shared voxels’ activity according to the perceived fairness of the people shown in the stimuli. Concretely, this was implemented by looking within the execution mask for voxels surviving the contrast (Action Movies—Control Movies) _fair_—(Action Movies—Control Movies)_unfair_. This contrast resulted in no activated areas at p < .05 with FDR multiple comparisons correction. Lowering the threshold to p < .005 uncorrected (minimum cluster size 10) identified one cluster of 11 voxels located in the prefrontal region of the thalamus. Looking outside the shared circuits by removing the execution mask (thresholded at p < .005 uncorrected) added the thalamic cluster (12 voxels; Fig 5 and Table 4). Inverting the GLM contrast ((Action Movies—Control Movies) _unfair_—(Action Movies—Control Movies)_fair_) also did not reveal any significant voxels falling within the execution mask at p < .05 with FDR correction. At the permissive p < .005, uncorrected (minimum cluster size 10) threshold, two clusters were identified, one in Area 2 on the right (26 voxels) and one in Area 6 on the left (24 voxels). Removing the execution mask and relaxing the threshold to p < .005 (uncorrected) revealed several clusters, shown in Fig 5 and listed in Table 4. These clusters do not overlap with those found in the MVPA role classification, as shown in Fig F in S1 Text.

**Table 4:** Activated voxels for the contrast. 1) (Action Movies—Control Movies) fair—(Action Movies—Control Movies)unfair, not masked with execution (p < .005 uncorrected, min cluster size 10 voxels) and 2) (Action Movies—Control Movies)unfair—(Action Movies—Control Movies) fair, not masked with execution (p < .005 uncorrected, min cluster size 10 voxels). For each cluster: size k in voxels and for the local maxima within each cluster: corresponding t value, MNI coordinates (x, y, z) in mm, hemisphere (R: right, L: left), anatomical localization (Brodmann Area, BA), and cytoarchitectonic localization (as given by the SPM Anatomy toolbox) when available. z-scores were calculated using the function from [46], transforming the SPM t-scores into z-scores for comparison with Singer et al., 2006.

**A**								
Size (voxels)	BA	Hemisphere	Anatomical Area	x	y	z	t	z-score
12		L	Prefrontal Thalamus	-16	-6	10	4.70	3.58
**B**								
Size (voxels)	BA	Hemisphere	Anatomical Area	x	y	z	t	z-score
150		R	Posterior Cingulate Cortex	10	-40	30	5.54	3.97
		R	Precuneus	14	-48	22	5.38	3.90
58		L	Middle Frontal Gyrus	-26	18	40	4.70	3.58
54		L		-2	0	-8	4.24	3.34
		R		4	2	-8	3.82	3.11
49		R	IPC(PGa)	40	-46	12	5.57	3.98
48		L	Anterior Cingulate Cortex	-2	2	30	4.75	3.61
		R	Middle Cingulate Cortex	6	4	32	4.05	3.24
30		L	Superior Frontal Gyrus	-14	24	38	3.67	3.02
28	4p	L		-30	-32	40	5.06	3.75
	3a	L	Postcentral Gyrus	-32	-32	48	3.17	2.71
27	2	R		20	-44	54	4.87	3.66
27	3a	L		-18	-34	50	4.91	3.68
26		L	Middle Temporal Gyrus	-46	-60	18	3.75	3.07
24		L	Area 6	-34	-14	56	5.59	3.98
22		L	Superior Medial Gyrus	-8	42	44	3.70	3.04
21		L	Posterior Cingulate Cortex	-10	-44	20	4.05	3.24
19		L	Precuneus	-2	-52	18	3.31	2.80
16		L	Middle Temporal Gyrus	-60	-4	-14	5.04	3.74
15	7p	R	Precuneus	16	-64	48	4.02	3.22
12	17	R		30	-70	10	3.53	2.94
10		L	Middle Orbital Gyrus	-36	44	-6	4.88	3.67

## Discussion

Drawing from the observation that, in men, activity in regions involved in the experience of pain differed when viewing the pain of fair and unfair individuals [22], in this study we tested the hypothesis that shared circuits activity would vary depending on whether a fair or unfair individual performed goal-directed hand actions. We manipulated how fair participants found two confederates by staging a game between the participant and two confederates, one of whom played fairly and the other unfairly. Directly following the game session we measured brain activity using fMRI while participants first observed actions performed by the two confederates, and then performed actions themselves. Drawing from the results of both mass-univariate and multivariate analyses, we conclude that brain activity during action observation may be modulated by perceived fairness, but such modulation is subtle; robust activity is associated with observing the actions of both fair and unfair individuals.

The game procedure provoked the desired social interactions and judgments: participants adjusted their playing style to each confederate, rated the unfair confederate as less reasonable, pleasant, fair, and liked than the fair confederate, and described the confederates differently at debriefing. All participants were able to identify each confederate by color and role (fairness) at debriefing; indeed, many participants spontaneously referred to the confederates by color when describing their experience during the game session (e.g. one participant reported that he “tried to get more money when playing orange, then minimize damage with blue”). The strength and consistency of most participants’ descriptions of the confederates suggests that the manipulation was effective at producing the desired social responses.

Observation of the visual stimuli was associated with activity in the expected areas: strong activation of regions involved in executing hand actions while viewing the confederates’ hand actions (compared to the control stimuli) in the mass-univariate analyses, and highly accurate classification of stimulus type (control or action movie) in the MVPA. Compared to these robust effects, modulation of brain activity associated with observing the actions of the fair and unfair confederate was subtle: mass-univariate analysis identified several clusters with more activity for unfair actors, two of which also fell into the motor execution localizer, but only at a threshold of p < .005, uncorrected. The MVPA classified role significantly in the right hemisphere PFhIP ROI, even after Bonferroni multiple-comparisons correction for 6 ROIs. In addition, a searchlight MVPA revealed six clusters with significant classification at p< .005, uncorrected.

Clusters identified by the MVPA as informative for confederate fairness, though not meeting strict statistical thresholds, tend to fall into areas frequently associated with social cognition. For example, the middle cingulate cortex is thought to be related to emotional processing [12, 47], while the MTG, postcentral gyrus, and IPL have been repeatedly shown to be activated both while observing and execution actions [2, 3, 48]. However, within these regions, the searchlight results fell at the fringe of the locations most associated with action observation: the role-discriminating MTG and postcentral searchlights were more anterior than those typically found with increased BOLD during action observation and execution [2, 3, 48]. The IPL is particularly interesting, as it was significantly informative as an a priori anatomic ROI, not only suggested by the searchlight analysis results. The IPL has been shown to contain mirror neurons [49, 50], and its activity is somatotopically organized in humans [30, 51]. However, the IPL is involved in a substantial number of other functions relating to attention, agency and motor control [52], so the information in this region could reflect role-modulated changes in vigilance or attention, rather than motor simulation.

Why is it that activity associated with the pain of others has been reported to be modulated by perceived fairness, while we found such subtle indications of modulation associated with the actions of others? One factor is simply that fMRI analysis and interpretation has evolved over time: our statistical thresholds are more stringent than those of Singer et al. [22], causing us to consider findings less significant, despite effect sizes that are actually similar. Experiments in which participants see both the pain and the actions of others in the same paradigm will be needed to directly assess whether fairness modulates the representation of the pain of others more than, or as much as, those of the actions of others. Theoretically, a plausible explanation for a difference in fairness modulation between pain and action representations could derive from the different functions ascribed to these two systems. Many suggest that activations in the sensorimotor system serve to help the brain perceive [53–55] and predict the actions of others [17, 56–58]. Perceiving and predicting the actions of others is important irrespectively of whether we engage in collaborative or competitive interactions, and hence should only be minimally influenced by perceived fairness. On the other hand, activations in the aIns and ACC while observing the pain of others has been suggested to trigger helping behavior [59]. Helping behavior is adaptive in collaborative but not competitive settings, and should thus be modulated more strongly by perceived fairness.

## Supporting Information

S1 Text Supplemental text, tables, and figures. (DOCX)
