# Supplementary material for: Is Brain Activity during Action Observation Modulated by the Perceived Fairness of the Actor?
Source: PLoS One. 2016 Jan 28;11(1):e0145350. doi: 10.1371/journal.pone.0145350 (PMC4731574; doi:10.1371/journal.pone.0145350)
Supplement: S1 Text — (DOCX) [file pone.0145350.s001.docx]

|  |  | **C** | **D** |  | **C** | **D** |  | **C** | **D** |  | **C** | **D** |  | **C** | **D** |  | **C** | **D** |
| --- | --- | --- | --- | --- | --- | --- | --- | --- | --- | --- | --- | --- | --- | --- | --- | --- | --- | --- |
| 1 |  |  |  |  |  |  |  |  |  |  |  |  |  |  |  |  |  |  |
| 2 |  |  |  |  |  |  |  |  |  |  |  |  |  |  |  |  |  |  |
| 3 |  |  |  |  |  |  |  |  |  |  |  |  |  |  |  |  |  |  |
| 4 |  |  |  |  |  |  |  |  |  |  |  |  |  |  |  |  |  |  |
| 5 |  |  |  |  |  |  |  |  |  |  |  |  |  |  |  |  |  |  |
| 6 |  |  |  |  |  |  |  |  |  |  |  |  |  |  |  |  |  |  |
| 7 |  |  |  |  |  |  |  |  |  |  |  |  |  |  |  |  |  |  |
| 8 |  |  |  |  |  |  |  |  |  |  |  |  |  |  |  |  |  |  |
| 9 |  |  |  |  |  |  |  |  |  |  |  |  |  |  |  |  |  |  |
| 10 |  |  |  |  |  |  |  |  |  |  |  |  |  |  |  |  |  |  |
| 11 |  |  |  |  |  |  |  |  |  |  |  |  |  |  |  |  |  |  |
| 12 |  |  |  |  |  |  |  |  |  |  |  |  |  |  |  |  |  |  |
| 13 |  |  |  |  |  |  |  |  |  |  |  |  |  |  |  |  |  |  |
| 14 |  |  |  |  |  |  |  |  |  |  |  |  |  |  |  |  |  |  |
| 15 |  |  |  |  |  |  |  |  |  |  |  |  |  |  |  |  |  |  |
| 16 |  |  |  |  |  |  |  |  |  |  |  |  |  |  |  |  |  |  |
| 17 |  |  |  |  |  |  |  |  |  |  |  |  |  |  |  |  |  |  |
| 18 |  |  |  |  |  |  |  |  |  |  |  |  |  |  |  |  |  |  |
| 19 |  |  |  |  |  |  |  |  |  |  |  |  |  |  |  |  |  |  |
| 20 |  |  |  |  |  |  |  |  |  |  |  |  |  |  |  |  |  |  |
| 21 |  |  |  |  |  |  |  |  |  |  |  |  |  |  |  |  |  |  |
| 22 |  |  |  |  |  |  |  |  |  |  |  |  |  |  |  |  |  |  |
| 23 |  |  |  |  |  |  |  |  |  |  |  |  |  |  |  |  |  |  |
| 24 |  |  |  |  |  |  |  |  |  |  |  |  |  |  |  |  |  |  |
| 25 |  |  |  |  |  |  |  |  |  |  |  |  |  |  |  |  |  |  |
| 26 |  |  |  |  |  |  |  |  |  |  |  |  |  |  |  |  |  |  |
| 27 |  |  |  |  |  |  |  |  |  |  |  |  |  |  |  |  |  |  |
| 28 |  |  |  |  |  |  |  |  |  |  |  |  |  |  |  |  |  |  |
| 29 |  |  |  |  |  |  |  |  |  |  |  |  |  |  |  |  |  |  |
| 30 |  |  |  |  |  |  |  |  |  |  |  |  |  |  |  |  |  |  |
| 31 |  |  |  |  |  |  |  |  |  |  |  |  |  |  |  |  |  |  |
| 32 |  |  |  |  |  |  |  |  |  |  |  |  |  |  |  |  |  |  |
| 33 |  |  |  |  |  |  |  |  |  |  |  |  |  |  |  |  |  |  |
| 34 |  |  |  |  |  |  |  |  |  |  |  |  |  |  |  |  |  |  |
| 35 |  |  |  |  |  |  |  |  |  |  |  |  |  |  |  |  |  |  |
| 36 |  |  |  |  |  |  |  |  |  |  |  |  |  |  |  |  |  |  |

Fig A. Sample response sheet used by the blue player during the game session. The player led (moved first) during games in which his color appeared, while the striping in each column indicates his partner for that round. In this case the blue player partnered the green for his first round of games and the orange for his second.


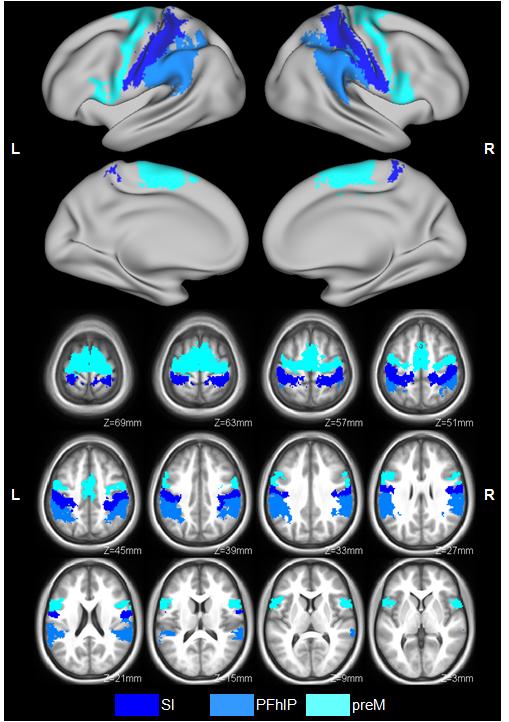


Fig B. The anatomical ROIs used in the MVPA; see also Table 1. The left and right hemisphere were defined separately in each ROI, but shown here in a single color.

blue player **Post-Game Questionnaire**

How reasonable was the orange player?

not at all ___|___|___|___|___|___|___|___ very

How reasonable was the green player?

not at all ___|___|___|___|___|___|___|___ very

How pleasant and agreeable was the orange player?

not at all ___|___|___|___|___|___|___|___ very

How pleasant and agreeable was the green player?

not at all ___|___|___|___|___|___|___|___ very

How much did you like the orange player?

not at all ___|___|___|___|___|___|___|___ very

How much did you like the green player?

not at all ___|___|___|___|___|___|___|___ very

How fair was the orange player?

not at all ___|___|___|___|___|___|___|___ very

How fair was the green player?

not at all ___|___|___|___|___|___|___|___ very

How easy was it to learn the rules of the game?

not at all ___|___|___|___|___|___|___|___ very

Did you find the three payout charts similar?

not at all ___|___|___|___|___|___|___|___ very

What strategy would you have liked to play?

Other comments?

Fig C. Example post-game questionnaire. This questionnaire was given to the blue player; three versions of the questionnaire were used, so that each player was asked to rate his two companions. The questionnaire was distributed after counting chips and announcing the game winner.


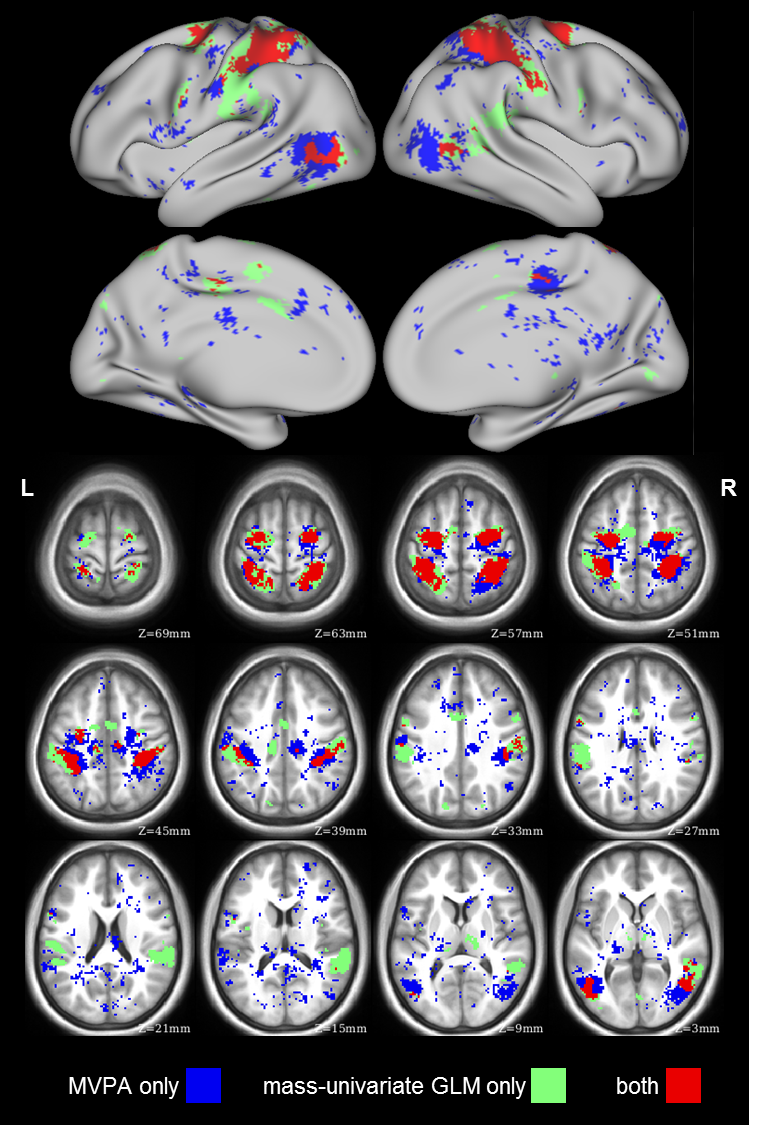


Fig D. The action observation network found by the two analytical methods is quite similar for the visual stimuli. Here, the MVPA searchlight accuracy map (from Fig 3) is plotted in blue (thresholded to only show voxels with accuracy above 0.65) and the shared voxels identified by the mass-univariate GLM (from Fig 2) is plotted in green (thresholded to only show voxels with t above 2.2). Voxels surviving both thresholds are plotted in red. Slice numbers are coordinates in mm.


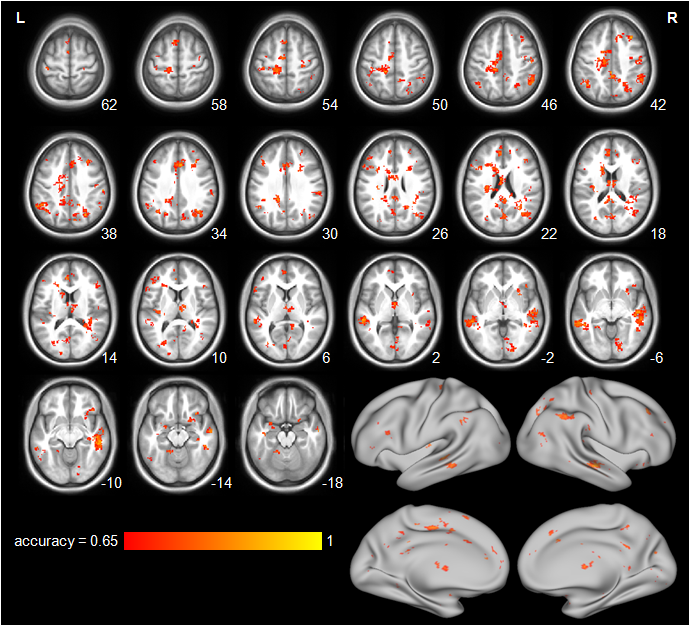


Fig E. Clusters in which confederate role could be classified in the searchlight MVPA, with more lenient thresholding than Fig 5 in the main text. These clusters were identified by taking voxels with p < 0.05 (uncorrected) in the permutation test, with a minimum cluster size of 20 voxels. Slice numbers are coordinates in mm.


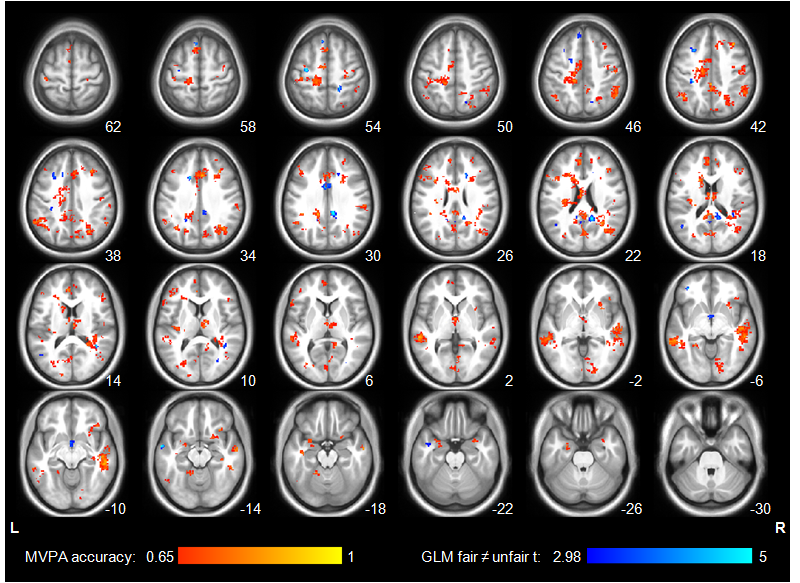


Fig F. The clusters distinguishing confederate role found by the mass-univariate analysis (blue) are not similar to those found by the searchlight MVPA (orange). The MVPA results here are the same as in Fig E, while the mass-univariate results are the same as those from Fig 5 in the main text. Slice numbers are coordinates in mm.

# Supplemental Methods

## Cover Story

This text was used to describe the experiment to people who responded to the advertising.

Thank you for your interest!

I am recruiting subjects for a two-part experiment, one about game playing strategies and one about how the brain responds to the sight and sound of people doing simple everyday actions (like lifting up a cup). "Reading" other people is very important in everyday life, and especially important in interactive strategy games like poker. We are interested in the relationship between how people play strategy games and how their brain reacts when they see other people's actions.

There are three appointments for the experiment: a preliminary appointment, a game-playing session, and fMRI scanning. All three parts will take place in Antonius Deusinglaan 2. The game is played in the morning by three subjects together, sitting around a table, with me there to give instructions and monitor the progress. During the fMRI scanning (later in the same day as the game session) you will see movies and hear sounds of the other subjects doing simple actions. During the preliminary appointment I will show you the mock MRI scanner (the opening is very small and some people become claustrophobic while being scanned) and record you performing simple actions (common everyday things). The films and sound recordings will be presented to the other subjects while they are in the scanner. I will give you a large shirt to wear over your clothes during the filming (so that everyone will look more similar), and your face will not be in the films (only your torso, arms, and hands). I will only use the recordings for the experiment - your name will never be on them.

You will receive compensation of 7.50 euro/hour, and the experiment will take about 4 hours, so it is 30 euros total. The winner of the game will receive a cash prize, with the amount depending on how many points are earned during the game. This amount will probably be less than 10 euros. You will receive the 7.50/hour during the game regardless of how well you do - the prize money is extra, and not guaranteed (three subjects play the game together, but only one of you can win).

I require that the subjects speak English, because I cannot speak Dutch very well, not because it is important for the experiment. So you do not need to be perfect, just able to understand the instructions and fill out some written questionnaires in English. If you can read this email it should be fine.

I am currently scheduling people for April. We need to schedule three different things: a preliminary appointment (an hour or less), the game playing session (an hour) and the scanning session (about two hours). If you are interested in participating we can talk on the phone so that I can answer your questions (and ask you some screening questions to make sure that the fMRI scanning will be safe for you; it is only dangerous if you have metal in your body) and schedule the appointments.

This description contained several deceptions: only one person was tested at a time, and each played the game with the same two confederates. The participant was always scanned immediately following the game session (not in the afternoon); the confederates were not scanned. The participant was filmed during the preliminary appointment, but the recordings were never viewed and were destroyed. Finally, the game playing strategies were not investigated.

## Game Session

The game was an alternating [1, 2] version of the Prisoner’s Dilemma game [e.g. 3, 4, 5], found during pilot testing to provoke the strongest impression of the confederates. The game was played with the participant, two confederates, and experimenter sitting around a table. Paper “choice sheets” were given to each player (Fig A), and a payout matrix (Table A) was placed in the center of the table in full view. The choice sheets had two columns for each round of games, marked “C” and “D”, in which the player marked his choice for a particular game. Each game was played alternating and openly: the leading player would mark his sheet in either the “C” or “D” column, announcing his choice, and then his partner would mark his sheet and verbally report his choice. After the partner’s choice the experimenter would distribute or take away poker chips from each player depending on the payout matrix being used for that round. The leading player alternated every three turns, so that each person moved first on half of the games in a round. The participants were told that “C” stood for “cooperate” and “D” for “defect,” but the name of the game was not given until debriefing and no context or scenario was provided.

The game was played by two people at a time, with the third watching. Each player stacked his chips on the table in front of him in clear view and was encouraged to check that the experimenter was scoring each player correctly. The players were not allowed to discuss the game or strategies during play. Three rounds of the game were played, each with a different payout matrix (Table A), such that penalties for mutual defection increased with each round. This provided more interest and challenge to the game, and strengthened the cover story that the experiment concerned game playing strategies. Each person played a round with each other person (i.e. participant vs. fair confederate; participant vs. unfair confederate; fair confederate vs. unfair confederate) on each payout matrix, for a total of nine rounds of games (of which each individual participated in six). The winner of the game was given a bonus monetary payment, with the amount of the bonus dependent upon how many chips he finished with (actual bonuses ranged between €6 and €10). The bonus payout scheme was announced in advance, and participants were asked to try to win the game, but also to maximize points. The number of games in each round was not announced, but was determined in advance by the experimenter and varied randomly from 24 to 36.

When the final game round was completed the experimenter recorded each players’ final number of chips, announced the winner and the bonus he received, and then distributed questionnaires (Fig C) to each player. The questionnaire was adapted from that used in Singer et al. [6] and consisted of four questions to determine the participants’ opinions of the confederates, an open-ended question to elicit comments, as well as questions about game strategy and rules, included to be consistent with the cover story that the study was about game playing strategies.

Table A. Prisoner’s Dilemma payout matrices used during the game session. The payout matrices were always played in order (1, then 2, then 3), with each person playing each other person once with each matrix.

| \| Matrix 1 \| \| first \| \| \| --- \| --- \| --- \| --- \| \|  \|  \| C \| D \| \| (second) \| (C) \| 2 (2) \| 3 (0) \| \| (D) \| 0 (3) \| 1 (1) \| | \| Matrix 2 \| \| first \| \| \| --- \| --- \| --- \| --- \| \|  \|  \| C \| D \| \| (second) \| (C) \| 2 (2) \| 4 (-1) \| \| (D) \| -1 (4) \| 0 (0) \| | \| Matrix 3 \| \| first \| \| \| --- \| --- \| --- \| --- \| \|  \|  \| C \| D \| \| (second) \| (C) \| 3 (3) \| 5 (-3) \| \| (D) \| -3 (5) \| -1 (-1) \| |
| --- | --- | --- | --- | --- | --- | --- | --- | --- | --- | --- | --- | --- | --- | --- | --- | --- | --- | --- | --- | --- | --- | --- | --- | --- | --- | --- | --- | --- | --- | --- | --- | --- | --- | --- | --- | --- | --- | --- | --- | --- | --- | --- | --- | --- | --- | --- | --- |

## MVPA Significance Testing

Estimating significance for the MVPA required somewhat unusual procedures, due to the necessity of leave-one-subject-out cross-validation. More often, MVPA are performed in each participant individually, and then these independent values (e.g., classification accuracy) are statistically combined for the group-level results (e.g., mean accuracy). The significance of that group-level statistic can then be established by parametric (e.g., a t-test) or permutation tests [7-9]. These methods are not appropriate for the current analysis, however, because we do not have independent estimates for each participant, but only an estimate for the group as a whole: the accuracy of the leave-one-subject-out cross-validation. Thus, the significance testing followed the procedures suggested in Etzel [8] for designs in which partitioning is on the subjects. Specifically, to maintain the dataset’s variance structure (examples nested within participants), each iteration of the permutation test was created by flipping the labels within the examples of one or more participants. For example, one valid permutation relabeling is to flip the confederate role labels on the third participant’s data, but no relabeling would result in both of the third participant’s examples being labeled as corresponding to the fair confederate. 16,383 such permutation relabelings are possible (_15_C_1_ + _15_C_2_ + _15_C_3_ + _15_C_4_ + _15_C_5_ + _15_C_6_ + _15_C_7_), of which 1000 were chosen at random, so the most significant p-value possible (true labeling more accurate than all permutation relabelings) is .001.

For multiple-comparisons correction, a simple Bonferroni correction was appropriate for the ROI-based MVPA: there are only six distinct, non-overlapping ROIs (three areas, each in both hemispheres; see Fig B). Such a straightforward method does not exist for the searchlight analysis, however. One difficulty is that adjacent searchlights are not independent (because they share many of the same voxels), and principled methods for adjusting the statistical threshold to reflect this spatial dependency do not exist. Permutation-based methods [9] for establishing cluster significance thresholds in searchlight analyses require the analyses to have been performed within each participant individually, and thus are not applicable to the current leave-one-subject-out design. Given that no validated multiple-comparisons correction for the searchlight results exists, rather than attempt an arbitrary and possibly misleading correction, we chose to present the searchlight results thresholded and clustered, but uncorrected for multiple comparisons, fully acknowledging that correction would be preferred. To assist the reader in evaluating the results, they are presented thresholded for searchlights with p < .01 (as determined from the permutation test, which was carried out within each searchlight individually), minimum cluster size of 50 voxels in the main text, and at the more lenient thresholds of p < .05, minimum cluster size 20 voxels in Fig E. Accordingly, while we think it is likely that at least some of the clusters shown in Fig 4 can truly distinguish confederate role in this dataset, we do not wish to oversell this claim, or imply more confidence than is appropriate.

Table B. Mean (standard deviation) of the responses to the post-game questionnaire. The fair confederate was ranked higher (more reasonable and pleasant) than the unfair on all four questions; the difference is significant (paired t-test) at p < .0001 for all questions.

|  | Fair Confederate | Unfair Confederate |
| --- | --- | --- |
| How reasonable? | 7.2 (1.1) | 2.7 (1.9) |
| How pleasant and agreeable? | 7.3 (.7) | 2.5 (1.1) |
| How much liked? | 6.9 (.9) | 3.1 (1.6) |
| How fair? | 6.7 (1.3) | 3.1 (1.6) |

# Supplemental References

1. Nowak MA, Sigmund K. The Alternating Prisoner's Dilemma. Journal of Theoretical Biology. 1994;168(2):219-26.

2. Frean MR. The Prisoner's Dilemma without Synchrony. Proceedings of the Royal Society of London Series B: Biological Sciences. 1994;257(1348):75-9. doi: 10.1098/rspb.1994.0096.

3. Axelrod R, Hamilton WD. The evolution of cooperation. Science. 1981;211(4489):1390-6. doi: 10.1126/science.7466396.

4. Doebeli M, Hauert C. Models of cooperation based on the Prisoner's Dilemma and the Snowdrift game. Ecology Letters. 2005;8(7):748-66.

5. Pruitt DG. Reward Structure and Cooperation: the Decomposed Prisoner's Dilemma Game. Journal of Personality and Social Psychology. 1967;7(1):21-7.

6. Singer T, Seymour B, O'Doherty JP, Stephan KE, Dolan RJ, Frith CD. Empathic neural responses are modulated by the perceived fairness of others. Nature. 2006;439(7075):466-9. PubMed PMID: 16421576.

7. Pereira F, Botvinick M. Information mapping with pattern classifiers: A comparative study. NeuroImage. 2011;56(2):476-96.

8. Etzel JA, editor MVPA Permutation Schemes: Permutation Testing for the Group Level. 5th International Workshop on Pattern Recognition in NeuroImaging (PRNI); 2015; Stanford University, USA: IEEE.

9. Stelzer J, Chen Y, Turner R. Statistical inference and multiple testing correction in classification-based multi-voxel pattern analysis (MVPA): Random permutations and cluster size control. NeuroImage. 2013;65:69-82.
